# Viscous Cervical Environment-on-a-Chip for Selecting High-Quality Sperm from Human Semen

**DOI:** 10.3390/biomedicines9101439

**Published:** 2021-10-10

**Authors:** Manhee Lee, Jin Woo Park, Dongwon Kim, Hyojeong Kwon, Min Jeong Cho, Eun Ji Lee, Tai Eun Shin, Dae Keun Kim, Seungki Lee, Do Gyeung Byeun, Jung Jae Ko, Jae Ho Lee, Jung Kyu Choi

**Affiliations:** 1 Department of Physics, Chungbuk National University, Cheongju 28644, Chungbuk, Korea; mlee@cbnu.ac.kr (M.L.); kdwon95@gmail.com (D.K.); 2 Department of Andrology, CHA Fertility Center Seoul Station, Jung-gu, Seoul 04637, Korea; bearjinwoo@chamc.co.kr (J.W.P.); jkk226@chamc.co.kr (H.K.); eunji1126@chamc.co.kr (E.J.L.); s7530254@chamc.co.kr (T.E.S.); 3 Department of Biomedical Science, College of Life Science, CHA University, Pochen 11160, Korea; mjjj0725@gmail.com; 4 Department of Urology, CHA Fertility Center Seoul Station, CHA University School of Medicine, Jung-gu, Seoul 04637, Korea; dkkim@cha.ac.kr; 5 Biological and Genetic Resources Assessment Division, National Institute of Biological Resources, Incheon 22689, Korea; metany@korea.kr; 6 Department of Biotechnology, College of Life and Applied Sciences, Yeungnam University, Gyeongsan 38541, Korea; qusehrudwkd173@gmail.com

**Keywords:** human sperm, polyvinylpyrrolidone (PVP), cervical mucus, sperm-sorting chip, motility, sperm-head vacuole, DNA integrity, micro-viscometry

## Abstract

When ejaculated sperm travels through the vagina to the uterus, mucus secreted by the cervical canal generally filters out sperm having low motility and poor morphology. To investigate this selection principle in vivo, we developed a microfluidic sperm-sorting chip with a viscous medium (polyvinylpyrrolidone: PVP) to imitate the biophysical environment mimic system of the human cervical canal. The material property of the PVP solution was tuned to the range of viscosities of cervical mucus using micro-viscometry. The selection of high-quality human sperm was experimentally evaluated in vitro and theoretically analyzed by the convection-diffusion mechanism. The convection flow is shown to be dominant at low viscosity of the medium used in the sperm-sorting chip when seeded with raw semen; hence, the raw semen containing sperm and debris convectively flow together with suppressed relative dispersions. Also, it was observed that the sperm selected via the chip not only had high motilities but also normal morphologies and high DNA integrity. Therefore, the biomimetic sperm-sorting chip with PVP medium is expected to improve male fertility by enabling the selection of high-quality sperm as well as uncovering pathways and regulatory mechanisms involved in sperm transport through the female reproductive tract for egg fertilization.

## 1. Introduction

Assisted reproductive technologies (ARTs) have helped in the birth of approximately 5 million babies since the first reported birth from in vitro fertilization (IVF) in 1978 in England, and these numbers are rapidly increasing each year [1]. Since then, ARTs such as IVF and intracytoplasmic sperm injection (ICSI) have become effective treatment options for infertile couples [2]. However, globally, an average of 48.5 million couples has still not succeeded in birthing children despite utilizing ARTs for durations exceeding 5 years, indicating a very limited birth rate in current clinical practice of ARTs. Male infertility accounts for approximately 30–50% of the total cases of infertility, with infertile men having increased tendencies for abnormal morphologies, low concentrations, poor motilities, and elevated levels of DNA damage in their sperm [3]. Thus, it is important to screen for high-quality sperm from raw semen samples of infertile men to achieve successful pregnancies in ARTs.

It has been reported in literature that fertilization with sperm of poor competence might induce aneuploidies in the preimplantation embryos [4]. However, the conventional swim-up or density gradient centrifugation methods used in ARTs for selection of high-quality sperm from raw semen may increase the probability of DNA damage during the repetitive centrifugation steps, which also typically entail multiple cumbersome, time-intensive processes, and unnatural procedures [5]. To address these problems, microfluidic sperm-sorting chips (SSCs) were recently developed for selection of high-quality sperm through integrated research in reproductive medicine and microfluidic technology [6]. Microfluidic chips provide a spatial microenvironment that imitates the female reproductive tract in their microchannels and have demonstrated the capability to sort motile and morphologically normal sperm cells from raw semen based on the principles of fluid dynamics, without centrifugation [7,8]. In addition, the SSC yielded higher DNA normality of sperm than the conventional swim-up methods; the swim-up method involves the repeated centrifugations, which causes considerable damage to the DNA of sperm [9]. However, most SSCs with various designs of fluidic channels use low-viscosity media [10], in contrast to the naturally occurring human cervical mucus, which has high viscosity.

Cervical mucus is secreted in the cervix of the female reproductive tract and forms a viscous barrier to sperm in the process of fertilization of the egg. The cervical mucus, which is a mixture of fluids, ions, and compounds, is highly viscous and has been shown to play an important role in selecting healthy sperm from raw semen during normal fertilization in vivo [11,12]. However, the importance of the fluid viscosity in mammalian reproduction concerned with fertilization or ARTs has been largely overlooked. Recently, in Sinton’s group, sperm dynamics in viscous media composed of hyaluronic acid and/or methyl cellulose [13] was investigated, and motility changes were observed with changes in the environmental viscosities of these media [14]. Although the linearity between sperm motion and viscosity of the medium was shown, the fundamental mechanism behind such a behavior is yet to be understood [15]. In addition, very little is known about how the motilities of sperms are directly related to the other important properties of reproduction, such as sperm-head morphology and DNA integrity.

In this paper, we present an SSC with polyvinylpyrrolidone (PVP) solution as the viscous medium, which provides an environment similar to cervical mucus in vivo. Natural sperm selection processes in the cervical mucus based on sperm motility in vivo were experimentally demonstrated using the SSC with PVP medium and theoretically analyzed via numerical simulations. Furthermore, we observed that the highly motile sperm selected using the SSC showed normal morphologies and high DNA integrity. These results could thus be used to understand the fundamental mechanisms of natural selection of high-quality sperm in vivo as well as to gain insight into how spermatozoa navigates to the egg for fertilization in the female reproductive tract.

## 2. Materials and Methods

### 2.1. Sample Collection and Preparation of Sperm Suspension

We used human semen samples collected from men undergoing evaluations for infertility after at least 3 days of abstinence; further, informed consent was obtained from each participant. All research activities involving the human sperm samples were reviewed and approved by the Institutional Review Board (IRB) of CHA University (1044 308-201701-BR-003-03; 01-May-2020). After liquefaction, the raw semen samples were assessed according to WHO guidelines, with the criteria for level +4 debris, including RBCs or WBCs, immotile sperm, and epithelial cells [16].

### 2.2. Fabrication of Sperm-Sorting Chip

Two pieces of poly(methyl methacrylate) (PMMA) of dimensions 18 × 16 mm were cut using a laser cutter (MYL-0705, Seoul, South Korea) to fabricate the SSC. The design for the SSC was first generated using a CAD program and implemented with RDWORKSV8 software for cutting. First, the inlet and outlet ports with diameters of 2.2 and 1 mm, respectively, were created by the laser cutter. Second, to create the microfluidic channel simulating the passage of sperm, a semielliptical section of 1 mm width and 1.2 mm thickness was cut by the laser. Consecutively, the microfluidic channel of the SSC was created by applying polydimethylsiloxane (PDMS; Dow Corning) as an adhesive to both surfaces of two PMMA substrates. Finally, the PMMA chip was attached to a petri dish by using the PDMS. The chip was then placed on a 60 mm petri dish, which was maintained at 65 °C for 5 h to cure the PDMS. To ensure that the entire assembly was sterile, all components were washed with 70% ethanol, air dried, and assembled inside a cell-culture hood.

### 2.3. Purification of the Sperm Samples and Analyses of Sperm Motilities, Velocities, and Morphologies

**Figure 1:**
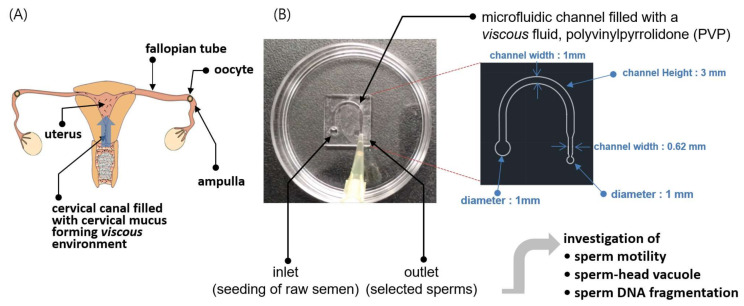
Schematic illustration of the methodology of the sperm-sorting chip (SSC). (**A**) Ejaculated sperm pass through the vagina and cervical canal and approach the ampulla (fertilization site) of the fallopian tube through the uterus. The cervical mucus is a fluid secreted in the cervical canal that filters out sperm with poor morphologies and low motilities as they pass into the uterus. (**B**) An SSC was developed to select high-quality sperm in vitro using the microfluidic technique, in which a viscous polyvinylpyrrolidone (PVP)-based solution was used as the medium to imitate the viscous environment of cervical mucus in vivo. The viscosity of the PVP medium was tuned to that of the cervical mucus via micro-viscometry. The sperm selected by the SSC were experimentally analyzed for motility, head vacuole, and DNA fragmentation as well as theoretically assessed via numerical simulations.

We used semen samples from 10 different persons, and the data were collected from triplicate experiments for each sample. The sperm was analyzed with WHO criteria, as described in Supplementary Material III. To purify the sperm samples, about 60 μL of either 1.5% or 3% PVP (PVP clinical grade, 10905000 Origio Coopersurgical DM) was injected at the channel inlet and fully loaded within the channel of the SSC (Figure 1B). The control medium was prepared using SAGE solution (sperm wash medium, QUINN’S^®^ Sperm Washing Medium, ART-1005, Coopersurgial DM) only, without any added PVP. Then, about 10 μL of raw semen samples were loaded at the seeding point of the SSC individually and incubated at 37 °C with 5% CO_2_. We then tracked the sperm cells and observed the patterns of sperm motility and velocity at the end of the channel for 40 s once every 10 min up to 40 min, using an inverted microscope (Eclipse Ti2; Nikon, Tokyo, Japan) equipped with a camera (DS-Ri2; NIKON) and imaging software (NIS-Elements ver. 4.4.; NIKON). At the outlet, we investigated the sperm-head vacuoles in each sample using high magnification (6100×) microscopy (Eclipse Ti2-E; NIKON) with high-power differential interference contrast (DIC) optics.

We prepared the control sperm group with swim-up methods, which is routine sperm preparation protocol for in vitro fertilization. Raw sperm liquefaction was done for 20~30 min at room temperature. Then, semen was mixed well with sperm washing media and centrifugated for 10 min at 1500 rpm. After centrifugation, the supernatant was removed and fresh media was carefully added to the sperm pellet. A 45° angle posited tube was kept at 37 °C for 30 min in 5% CO_2_. Finally, motile sperm in the media layer was harvested for the analysis.

### 2.4. Evaluation of Sperm DNA Fragmentation

Sperm DNA fragmentation is generally an indication of abnormal genetic material in the sperm. The sperm DNA fragmentation index (DFI) reflects the integrity of and damage to the sperm DNA and is widely regarded as a key parameter for assessing male fertility; this parameter was used to evaluate DNA integrity using the halosperm kit (halosperm G2 HT-HSG2, Madrid, Spain), according to the manufacturer’s protocol. The halosperm kit is based on the sperm chromatin dispersion (SCD) technique, where normal sperm form halos with the loops of the DNA at the sperm head; note that these will not be present in cells with damaged DNA. The level of DNA fragmentation was estimated by the halo size using an inverted microscope equipped with a DS-5i camera (Eclipse Ti-U; Nikon, Tokyo, Japan) with NIS-Elements Viewer Imaging Software version 4.6 (Nikon, Tokyo, Japan).

### 2.5. Numerical Simulation of Sperm Dynamics

We used a formalism developed by Fisher et al. [17] to investigate the sperm dynamics, where sperm motion was described as an active matter with enhanced Brownian motion (see Equations (1) and (2) in the text). To numerically solve Equations (1) and (2), we discretize the equations of motion as follows:

(M1) $$x_{n+1}=x_{n}+v_{0}\cos\theta_{n}\;dt+V_{x}\;dt,$$

(M2) $$y_{n+1}=y_{n}+v_{0}\;\sin\theta_{n}dt,$$

(M3) $$\theta_{n+1}=\theta_{n}+\sqrt{2D_{r}}\;\bar{\zeta }\;\sqrt{dt},$$

where $\bar{\zeta }\;$ is the Gaussian random variable with zero mean and unit variance. We used the software Mathematica to solve Equations (M1)–(M3) with the initial condition $x_{0}$ = $x_{0}$ = $\theta_{0}$ = 0 and time interval d*t* = 0.001 s for different rotational diffusion coefficients: $D_{r}$ = 0.2, 0.1, 0.05, and 0.02 rad/s. The stochastic Equations (M1)–(M3) indicate that a sperm moves in a new random direction at each time step d*t* by a fixed distance $v_{0}$d*t*, resulting in enhanced Brownian motion. The motion is observed to be highly linear progressive at a low rotational diffusion constant $D_{r}$, whereas the motion becomes randomly diffusive at a high $D_{r}$.

### 2.6. Statistical Analysis

All data are expressed in terms of the means ± standard error of the mean in triplicate measurements. The statistical analyses were performed with Statistical Package for the Social Science (SPSS), in which one-way ANOVA analysis was used, and the significant differences are indicated by asterisks (* *p* < 0.05).

## 3. Results and Discussion

The overall research objective for the proposed SSC is schematically depicted in Figure 1. The female reproductive tract is represented in Figure 1A; from the vagina to cervix, followed by a semielliptic path to the fallopian tube through the uterus. The tract through which the sperm pass gradually narrows as the sperm approach the oocyte. Cervical mucus has been known to play an important role in the selection of strong progressive sperm and filtering out those with poor morphologies and motilities (Figure 1A). We created a miniaturized semielliptic SSC with PVP as the viscous medium to imitate the environment of the cervical canal (Figure 1B). To achieve a viscous environment similar to that of the cervical canal for the sperm in the SSC, a mixture of PVP and sperm washing solution (SAGE, see Section 2.3) was prepared as the medium. Further, the mixing ratio was tuned via micro-viscometry such that the viscosity of the medium was similar to that of cervical mucus. Then, a drop of raw semen was seeded at the inlet of the SSC, and a drop of the sorted solution was collected at the outlet after approximately 40 min; this solution was subsequently investigated for sperm motility, head vacuole, and DNA fragmentation. Theoretical investigations were also performed on why highly motile sperm were selected in the high-viscosity condition by the SSC.

We prepared different sperm-sorting media with desired viscosities by mixing PVP and SAGE solutions (see Section 2.3) and measuring with a home-built micro-viscometer [18]. The viscosities of the media are controlled by changing the mixing ratio of the two solutions. Based on the measured viscosity values, the mixing ratio was tuned to obtain a medium with the final desired viscosity. Importantly, while conventional viscometers typically require a few milliliters volume of the target fluid for viscosity measurement, our micro-viscometer requires only a few microliters volume of fluid; this allows measurement of samples with very low volumes of about 10 µL.

**Figure 2:**
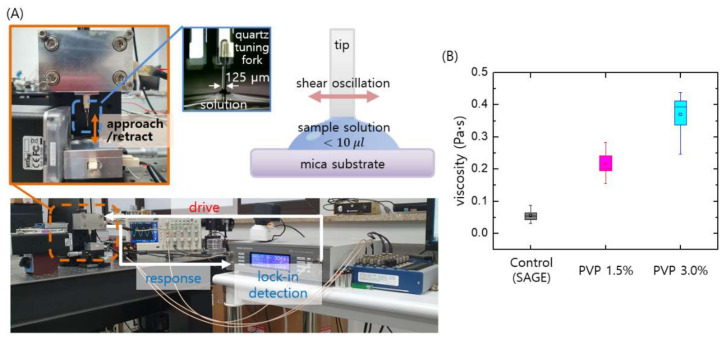
Micro-viscometry of the media used in the microfluidic SSC. (**A**) A home-built micro-viscometer is used to quantitatively measure the media viscosities, which are controlled by changing the mixing ratios of SAGE (sperm washing solution) and PVP. The tip shearing the fluid is harmonically driven, and its response is detected by the lock-in technique to measure the amplitude and phase of the tip oscillation. From the information of oscillation amplitude and phase shift of the shearing tip, the interaction force with the fluid and thus the fluid viscosity is quantitatively determined. (**B**) Three media solutions were prepared, namely the base solution (SAGE, the control) with a viscosity of about 0.07 Pa·s and two PVP solutions (1.5% and 3%) with relatively high viscosities in the range of 0.2–0.4 Pa·s, as measured using the micro-viscometer.

Figure 2A shows the home-built micro-viscometer based on a quartz tuning fork (QTF) sensor [18]. Tuning forks are widely used as force sensors in atomic force microscopes (AFMs), so we employed this sensor to construct the viscometer. A cleaved optical fiber with a flat bottom was used for the probing tip, and the tip was attached to one prong of the QTF to shear the fluid surface. When the tip gently touches the sessile drop of sample, the shear interaction with the fluid is measured quantitatively using dynamic force spectroscopy (DFS) with the AFM (see Supplementary Information for details) [19,20,21]. The DFS method allows determination of the elastic and damping coefficients of the interactions with the sheared fluid, and the viscosity η of the fluid is determined as η = 2 k_int_^2^/(σ^2^ ω^3^ ρ), where k_int_ is the elastic coefficient, σ is the interaction area (bottom area of the tip), ω is the angular frequency, and ρ is the density of the fluid. Note that the elastic coefficient k_int of the interaction is only used to obtain the fluid viscosity, thereby circumventing fluid–air interfacial effects [16]. As shown in Figure 2B, three media solutions were prepared, namely the base medium (SAGE, the Control) with viscosity about 0.07 Pa·s, and two PVP solutions with different concentrations (i.e., 1.5% and 3%) with their viscosities ranging from 0.2 Pa·s to 0.4 Pa·s to mimic the natural viscosity of cervical mucus in vivo, 0.1–1 Pa·s [14,22]. As shown later, the two PVP solutions (1.5% and 3%) enable the isolation and selection of highly motile sperm in the proposed microfluidic SSC.

**Figure 3:**
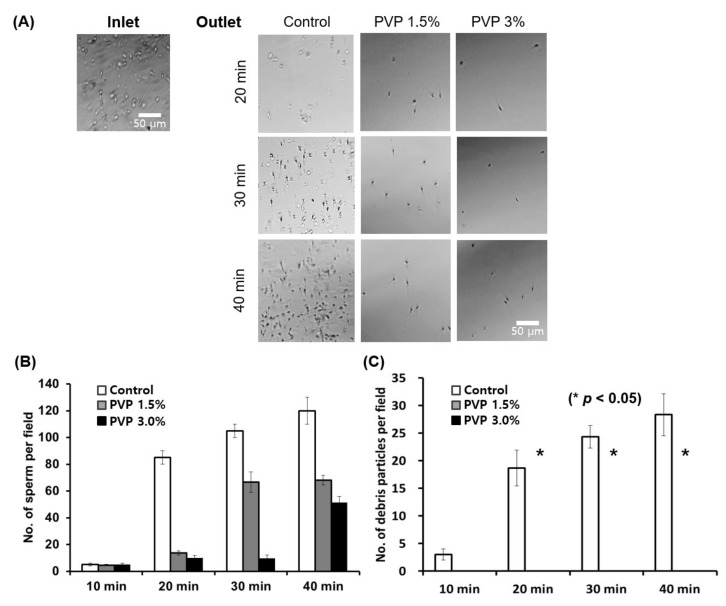
Quantitative analyses of sperm and debris isolated using the SSC for various PVP concentrations (0, 1.5%, and 3%) and incubation times. (**A**) Optical microscope images of the sorted sperm using the medium (45 µL) with PVP concentrations of 0 (Control), 1.5%, and 3%, after incubation for 20 min, 30 min, and 40 min at 37 °C in 5% CO_2_. (**B**) Sperm and (**C**) debris particle counts per field for the control, PVP 1.5%, and PVP 3% media every 10 min at the outlet of the chip. All data are expressed as means ± standard error of the mean (SEM) for triplicate measurements. The significant differences are indicated by asterisks (* *p* < 0.05 against control).

The SSC loaded with 1.5% and 3% PVP solutions enabled separation of the motile sperm cells at the outlet of the chip within 40 min, with complete removal of debris particles, immotile sperm, white blood cells (WBCs), and red blood cells (RBCs) from the human semen, as shown in Figure 3A. Notice that the number of sperm at the outlet first increases and then becomes constant with time. The initial injection of the semen into the inlet induces a flow in the channel until the hydrostatic pressure at the inlet is balanced with the outlet pressure. Since the flow rate decreases with the increase of viscosity, it takes a long time to achieve the inlet–outlet pressure balance for highly viscous medium. Therefore, the time when the number of sperm is saturated increases with the medium viscosity, as observed in Figure 3A. The SSC loaded with PVP 1.5% was demonstrated to successfully isolate the highest number of sperm cells at the outlet compared with the 3% PVP-loaded chip; however, with the control medium, both motile sperm and debris were observed at the outlet (Figure 3B,C). These results suggest that sperm separation might occur in viscous media in vivo, wherein the cervical mucus acts as the viscous barrier to separate the motile sperms. We note herein that the conventional swim-up sorting method does not completely remove WBCs from raw semen. The WBCs in semen can produce detectable amounts of reactive oxygen species (ROS), which may then cause nuclear and mitochondrial DNA damage in the sperm by inhibiting intracellular adenosine triphosphate (ATP) production. This improper ATP production in turn affects sperm motility [23]. In the proposed method, only the motile sperms are isolated, and the WBCs are completely removed from the semen.

**Figure 4:**
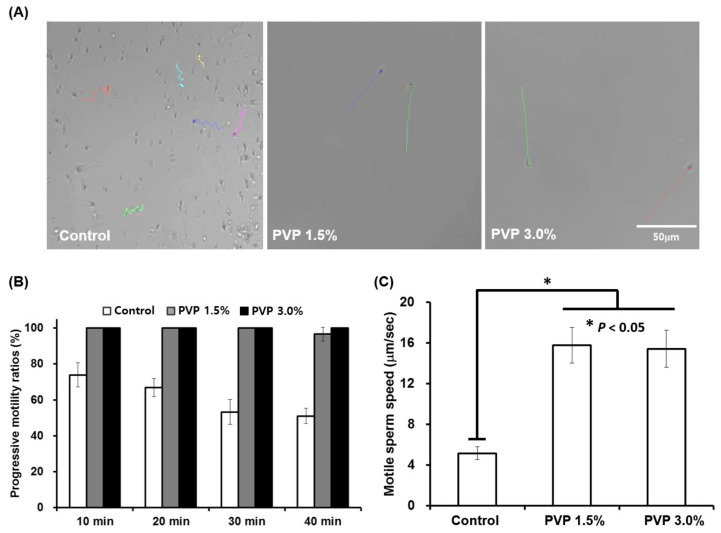
Motility analysis of sperm selected by the SSC for different PVP concentrations of 0, 1.5%, and 3%. (**A**) Real-time tracking of sperm at the outlet of the SSC, showing different progression patterns depending on the PVP concentrations (colored lines). (**B**) Progressive motility ratios in the control (0%), 1.5%, and 3% PVP media measured every 10 min at the outlet of the SSC. (**C**) Sperm velocities measured at the outlet of the SSC for the control, 1.5%, and 3% PVP media. All data are expressed as means ± SEM for triplicate measurements. The significant differences are indicated by asterisks (* *p* < 0.05 against control); scale bar: 50 µm.

The 1.5% and 3% PVP media showed mostly linear progression patterns, as marked with the position tracking curves (colored curves) in Figure 4A (see Supplementary Information for details). The linear progressive sperm motilities and velocities for both 1.5% and 3% PVP media were observed to be significantly different (*p* < 0.05) than those for the control group (Figure 4B,C). The viscous environment of the PVP solutions not only facilitate progressive motions of the sperm but also purify the sperm solution, acting as filters to remove immotile sperm, WBCs, and RBCs in the raw semen. The linear progression motility and velocity of sperm are important factors in the process of egg fertilization by a sperm cell. These factors also explain the natural mechanism of sperm selection in vivo, where the cervical mucus provides a viscous barrier that only the highly motile sperm can penetrate, as demonstrated in our experiment in vitro. Therefore, the linear progression motility of sperm has been critically associated with fertilization of eggs and embryonic development [24].

**Figure 5:**
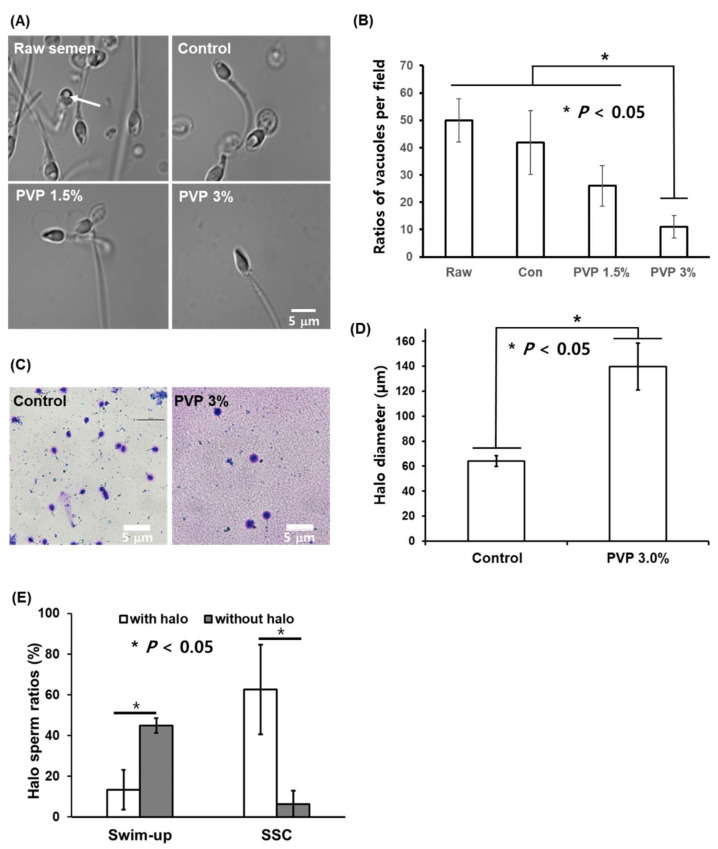
Sperm-head vacuoles of raw semen as well as control, 1.5%, and 3% PVP media observed under an optical microscope, and sperm DNA integrity. (**A**) Microscopic images of sperm in the control, 1.5%, and 3% PVP media under high magnification, where the arrow indicates a nuclear vacuole in the sperm head; scale bar: 5 µm. (**B**) Number of sperm with vacuole heads in the raw semen, control, 1.5% PVP, and 3% PVP based on microscope image analysis. (**C**) Evaluation of sperm DNA fragmentation using halosperm kit with a bright-field microscope and quantitative analysis of halo sizes between raw semen and 3% PVP. Human sperm stained using the halosperm kit were assessed by size measurements; sperm without DNA fragmentation showed large halos, whereas those with fragmented DNA showed smaller halos. scale bar: 5 µm (**D**) Halo sizes of sperm selected by the SSC with PVP 3% were greater than those with the control medium, indicating low DNA fragmentation. The significant differences are indicated by asterisks (* *p* < 0.05 against control). (**E**) Halo sperm ratios analysis for swim-up sperm and SSC sperm. The significant differences are indicated by asterisks (* *p* < 0.05 against control).

For the sperm selected by the SSC loaded with 1.5% and 3% PVP, the ratio of the number of sperm with vacuole head to sperm number was less than those for the semen and the control, as shown in Figure 5A,B. Statistically significant differences (*p* < 0.05) were observed in the numbers of sperm-head vacuoles among the experimental groups, with the 3% PVP medium exhibiting the lowest number among all groups (Figure 5B). This indicates that the sperm with vacuole head exhibited low motility, compared to the sperm with normal head. This observation is consistent with the density-gradient experiment [25], in which ‘top’ sperm shows a morphologically normal head and thus the motility of vacuole head sperm is lower than that of the normal head sperm. The sperm-head vacuoles are associated with chromatin condensation failure as well as sperm DNA damage [26,27,28]. The presence of sperm-head vacuoles has also been associated with abnormal sperm morphologies and has been reported to be deleterious to the outcomes of ARTs. Sperm with non-fragmented DNA selected by the SSC with 3% PVP were shown to produce large halos of dispersed DNA loops, whereas sperm with fragmented DNA have smaller halos in the control (Figure 5C). The halo sizes of the sperm selected by the SSC with PVP (139.8 µm) were significantly different (*p* < 0.05) than those in the control group (64.1 µm) (Figure 5D). Sperm cells with large halos have been reported to influence fertilization rate, embryo quality, and pregnancy in conventional IVF [29]. The SSC with PVP medium allows selection of sperm with DNA integrity required for normal embryonic development and live births. In addition, we investigated big halo sperm ratios for sperm sorted by the swim-up technique and SSC with 3% PVP. Figure 5E shows the SSC method significantly increased big halo sperm ratios (*p* < 0.05), compared to the swim-up method.

Among the different ARTs, ICSI is widely used in fertility clinics as a form of IVF, thus enabling fertilization by injection of a single sperm into an oocyte. The ICSI technology involves empirical skills on the part of the embryologist for the selection of a single sperm with normal head shape and high motility. The intracytoplasmic morphologically selected sperm injection (IMSI) method has also been used to select high-quality sperm without head vacuoles; this process requires expensive equipment as well as time-consuming and intensive labor to select the sperm cells, thus providing limited accessibility and affordability [30]. In contrast, the proposed SSC loaded with the PVP medium is shown to be a simple, fast, and efficient means of isolating highly motile sperm with normal head morphologies and high DNA integrity from raw semen. Further, this method minimizes the required hands-on experience. There exist different types of sperm-sorting chips based on microfluidics, which typically require additional instruments such as syringe pumps [31,32] and acoustic actuators [33]. However, our SSC is a standalone system. We use only the chip itself to sort out high-quality human sperm, which facilitates clinical usage.

In the future, we plan to use the high-quality sperm selected by the SSC to examine fertility rates and pregnancies by fertilizing human oocytes with the selected sperm via ICSI. The proposed SSC is an ICSI-dedicated chip that can select a small number of sperm of high quality, and the chip can be improved by altering the channel dimensions to isolate larger amounts of high-quality sperm for artificial insemination or IVF at fertility clinics.

The isolation of highly motile sperm using the SSC can be understood by the convection–diffusion mechanism [34] for active matter under sheared flows. The active matter model [35] was first employed by Fisher et al. for sperm cells, successfully explaining the individual and cooperative dynamics of sperm cells [36,37]. When the sperm solution is injected at the seeding point of the chip, a sudden increase at the inlet pressure drives the sperm cells into the microfluidic channel, with the cells undergoing movement via convection flow, until the inlet and outlet pressures are in equilibrium. The microfluidic channel is about 2.5 cm long in the lateral direction (here defined as x-axis), with width of about 1 mm (y-axis) and height of about 2.4 mm (z-axis). In other words, the sperm cells are highly confined in y- and z-axes, while the sperm cells are distributed throughout the fluid channel in the x-axis. Therefore, we consider a 2-dimensional motion of sperm cells in the x-y plan. The stochastic dynamics of a single sperm is described as (see Figure 4A):

(1) $$\frac{d\vec{r}}{dt}=v_{0}\hat{n}+V_{x},$$

(2) $$\frac{d\theta }{dt}=\zeta ,$$

where $v_{0}\hat{n}$ is the velocity vector of a sperm with speed $v_{0}$ and direction $\hat{n}$ = (cos *θ*, sin *θ*), $V_{x}$ is the instantaneous velocity of the transient convection flow, and *ζ* is the zero-mean delta-correlated random variable, ⟨*ζ*(*t*)*ζ*(*t*’)⟩ = 2$D_{r}$δ(*t* − *t*’), with rotational diffusion constant $D_{r}$. While the sperm is propelled with a velocity $v_{0}$ (Equation (1)), the direction of motion is subject to rotational diffusion (Equation (2)). This coupling between rotation and translation causes side-to-side movements across the directional axis. We note that the thermal translational diffusion is negligible compared with the self-driven motion, as indicated by the *Pelect* number *Pe*~104 for a motile sperm [37].

To numerically solve the stochastic equations of motion, Equations (1) and (2), we discretized the equations and solved them with relevant parameters (see Section 2). Herein, we assumed that the rotational diffusion constant, $D_{r}$, associated with rotational motion may depend on the viscosity of the environmental medium [34], whereas the progressive translational velocity $v_{0}$ would not vary much with viscosity [38]. For a colloidal sphere, the constant $D_{r}$ is inversely proportional to the viscosity [35], and this feature could be applied to sperm motion despite the geometrical complexity of the sperm. The exact value of $D_{r}$ for each sperm cell in a medium is difficult to determine, but the value of $D_{r}$ is expected to decrease as the viscosity of the medium increases. Thus, we use the rotational diffusion constant, which is here assumed to be inversely proportional to viscosity of the medium, as a model parameter for the sperm.

**Figure 6:**
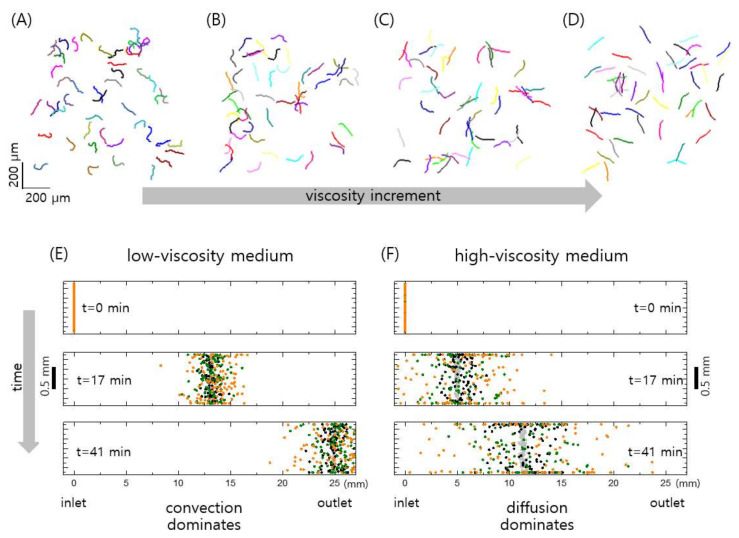
Theoretical description of sperm cell dynamics. (**A**–**D**) A sperm cell can be described as an active matter, self-propelled particle that is propelled with a speed of *v_0_* subject to rotational diffusion (see the text for detail). Trajectories over 10 s for 50 randomly distributed sperm with *v*0 = 15 μm/s are shown for different rotational diffusion coefficients *D_r_* = 0.2 (**A**), 0.1 (**B**), 0.05 (**C**), and 0.02 rad/s (**D**). (**E**,**F**) Spatial distributions of 100 sperm with speed *v*_0_ = 0.5 (grey dots), 1 (black dots), 5 (green dots), and 15 μm/s (orange dots) under shear flow in the channel filled with low-viscosity (*D_r_* = 0.1 rad/s) and high-viscosity (*D_r_* = 0.02 rad/s) media at different times t = 0, 17, and 41 mins.

Our model (Equations (1) and (2)) shows that the linearity of the sperm motion enhances as the medium viscosity increases, as shown in Figure 6A (see also Figure 4A, the experimental results). Essentially, the linearity of sperm motion is enhanced by the suppressed random rotation in a viscous medium. Since the random rotation is reduced at high viscosity medium, the trajectory of the sperm becomes straight in highly viscous medium. When the initial convection flow is diminished at the chip outlet, the sperm are purely self-propelled. To describe the self-propelled sperm at the outlet, we set $V_{x}$ = 0 in Equations (1) and (2). Figure 6A–D show the sperm trajectories obtained from Equations (1) and (2) with zero convention flow, $V_{x}$ = 0, for different rotational diffusion constants of $D_{r}$ = 0.2, 0.1, 0.05, and 0.02 rad/s. Notice that the rotational diffusion constant could be inversely proportional to the viscosity, i.e., $D_{r}$ ∝ 1/*η*. Thus, with the proportional constant 10^−2^ Pa, the diffusion constant $D_{r}$ = 0.2 rad/s corresponds to PVP viscosity 0.05 Pa·s, $D_{r}$ = 0.05 rad/s to 0.2 Pa·s, and $D_{r}$ = 0.02 rad/s to 0.4 Pa·s. The sperm motions in the high-viscosity medium, equivalently in low-rotational diffusion, are highly linear, compared to the motions in the low-viscosity medium, as consistently observed in our experiments (Figure 4A).

Furthermore, our model could explain how the viscous medium of human cervical mucus naturally allows selection of highly motile sperm. First, let us consider a low-viscosity medium, where convection flow dominates diffusion of the sperm; this results in spatial distributions of sperm that are overall similar to each other during the flow, despite different sperm speeds $v_{0}$ = 1, 5, and 15 μm/s, as shown in Figure 6E. Indeed, in our SSC (Figure 3A, the control), the motile and immotile sperm cells and debris convectively flowed toward the outlet with negligible relative dispersions. Therefore, it is difficult to select only motile sperm at the outlet of the channel that is filled with a low-viscosity medium.

Conversely, we can select motile sperm at the outlet when the channel is filled with a highly viscous medium (Figure 3A, PVP 1.5% and 3%). Specifically, the enhanced translational diffusion of the self-propelled sperm in viscous medium allows spatial isolation of highly motile sperm from raw semen, including motile and immotile sperm and debris (Figure 6F). In the highly viscous medium, the diffusion process dominates convection flow, and the self-propelled diffusion increases with the sperm speed $v_{0}$ in Equation (1). Therefore, the overall spatial distribution of sperm in the channel strongly depends on their speed rather than convection flow, which is suppressed in a viscous medium. The viscous media create a barrier through which only highly motile sperm can penetrate, and one can thus obtain sperm with high motility at the outlet of the SSC. The simple but robust microfluidic method presented herein resembles the in vivo environment of the cervical canal, which is filled with viscous mucus and enables natural selection of highly motile sperm for fertilization.

Our model of sperm motion, which is based on active matter dynamics, reveals a quantitative statistical behavior of the entire sperm volume, despite not describing the details of the motion of a single sperm, such as the movement of its flagellum [38]. Previous studies have shown the predominance of sperm motion in corners and near surfaces associated with the beating flagella [38], flagellar oscillation mechanisms [39], and the attraction and aggregation of sperm through hydrodynamic interactions [40]. While such descriptions of sperm motion allow understanding of the motion of a single or a few sperm, our approach enables us to describe the distribution of entire sperm in position and time. This allows to obtain the statistical yield of sperm with high mobility of about 2% at the outlet (Figure 6F), which might still be enough for use in ICSI of ART.

## 4. Conclusions

The proposed SSC loaded with polyvinylpyrrolidone (PVP) imitates the viscous environment of cervical mucus in the female reproductive system. Our PVP-loaded SSC allowed selection of highly motile sperm without any debris, and the selected sperm further showed linear progressive motilities with high speed. Moreover, the number of sperm-head vacuoles and amount of sperm DNA fragmentation were significantly decreased with both PVP media concentrations compared with the control. Our simulation study also showed that the sperm in highly viscous media exhibit highly linear motions because of the greatly reduced rotational motions. This directional motion of the sperm causes velocity-dependent translational diffusion, resulting in separation of motile sperms from raw semen containing motile and immotile sperm, different blood cells, and debris. These results exemplify the filtration principle for sperm in the cervical canal, where the viscous environment of the cervical mucus filters out sperm with poor motility, as demonstrated by our SSC with a viscous PVP medium in vitro. Based on these results, we intend to perform further clinical studies with human oocytes to examine the functionality of the sperm selected by the proposed SSC. The PVP solution has been generally used for sperm immobilization during ICSI in the clinics. However, potential harmful effects of PVP on sperm have been reported by Kato et al. [41]. In order to more safely use SSC, it is therefore necessary to develop new materials bio-compatible to sperm, oocyte, and embryo, which could replace the currently used PVP in the future. The presented SSC chip with a PVP medium could contribute to improving male fertility via high-quality sperm production as well as revealing the sperm navigation mechanisms in the female reproductive tract.

## Data Availability

Not applicable.

## Supplementary Materials

The Supplementary Materials are available online at https://www.mdpi.com/article/10.3390/biomedicines9101439/s1. In Supplementary Materials (SM), we provide the two videos of experiments, (I) micro-viscometry using our home-built setup and (II) sperm motion in three solutions with different viscosities observed by optical microscope (Figure 4A), and (III) Table of the information of 10 patients’ sperm used for our experiments. SM I (Video 1): Micro-viscometry of sperm sorting solutions. The video shows the quartz tuning fork with an optical fiber tip and the measured solution, SAGE. We release a drop of sample fluid on mica substrate, the tip approaches the fluid sample until the tip’s bottom gently touches the sample surface, and then retracts away from the sample until the fluid bridge is ruptured, while measuring the shear interaction between the tip and the fluid. From the measured shear force, we calculate the viscosity of the sample fluid. SM II (Video 2): Sperm motion in Control (PVP 0%), PVP 1.5%, and PVP 3.0% (Figure 4A in the main text). SM III (Table S1): The information of 10 patients’ sperm data used in our study.
