# Supplementary material for: Viscous Cervical Environment-on-a-Chip for Selecting High-Quality Sperm from Human Semen
Source: Biomedicines. 2021 Oct 10;9(10):1439. doi: 10.3390/biomedicines9101439 (PMC8533482; doi:10.3390/biomedicines9101439)
Supplement: Supplementary file 1 [file biomedicines-09-01439-s001.zip › biomedicines-1339251-supplementary.pdf]

Table 1 Characteristic of raw semen analysis

Patient	Sperm count (10^4)	Sperm motility (%)	Progressive motility (%)	Volume (ml)	Debris (*class)	WBC (10^6)
**1**	155	44	43	2.6	1	1.8
**2**	149	56	55	2.3	0	0.2
**3**	56	42	41	7.7	2	0.3
**4**	98	69	68	7.6	2	0.1
**5**	54	43	43	7.6	2	0.1
**6**	41	59	58	7.7	1	0
**7**	102	40	39	7.6	1	1
**8**	88	63	59	7.7	1	0.1
**9**	80	66	65	7.3	1	1
**10**	189	35	34	3.8	0	0

*class: Qualitative classification of debris concentration, where '0' corresponds to very low concentration of debris, '1' to medium concentration, and '2' to relatively high concentration of debris.
